# *Lactobacillus iners* Cell-Free Supernatant Enhances Biofilm Formation and Hyphal/Pseudohyphal Growth by *Candida albicans* Vaginal Isolates

**DOI:** 10.3390/microorganisms9122577

**Published:** 2021-12-13

**Authors:** Samuele Sabbatini, Sofia Visconti, Marco Gentili, Eleonora Lusenti, Emilia Nunzi, Simona Ronchetti, Stefano Perito, Roberta Gaziano, Claudia Monari

**Affiliations:** 1 Department of Medicine and Surgery, Medical Microbiology Section, University of Perugia, 06129 Perugia, Italy; samuele.sabbatini@gmail.com (S.S.); spallyvisco@gmail.com (S.V.); stefano.perito@unipg.it (S.P.); 2 Department of Medicine and Surgery, Pharmacology Section, University of Perugia, 06129 Perugia, Italy; marcogentili1988@hotmail.it (M.G.); eleo95@gmail.com (E.L.); simona.ronchetti@unipg.it (S.R.); 3 Department of Medicine and Surgery, University of Perugia, 06129 Perugia, Italy; emilia.nunzi@unipg.it; 4 Department of Experimental Medicine, University of Rome Tor Vergata, 00133 Rome, Italy; roberta.gaziano@uniroma2.it

**Keywords:** *C. albicans*, biofilm, *L. iners*, VVC, microbiota

## Abstract

*Candida albicans* is a commensal fungus of the vaginal mucosa and the principal etiological agent of vaginal candidiasis. Vaginal dysbiosis has been reported during vulvovaginal candidiasis (VVC), with a progressive decrease in *Lactobacillus crispatus* population and an increase in *L. iners* population. To date, the role of *L. iners* in VVC pathogenesis remains scarcely explored. Herein we investigated the in vitro effect of *L. iners* cell-free supernatant (CFS) on the ability of *C. albicans* to form biofilms. Biomass and metabolic activity were measured by crystal violet and XTT assays. Further, light microscopy was performed to determine the effect of *L. iners* CFS on biofilm cellular morphology. We found that *L. iners* CFS induced a significant increase in biofilm formation by *C. albicans* clinical isolates which were categorized as moderate or weak biofilm producers. This effect was associated with an enhancement of hyphal/pseudohyphal growth, and the expression levels of *HWP1* and *ECE1*, which are typical hyphae-associated genes, were upregulated. Overall, these results suggest that *L. iners* contributes to the pathogenesis of VVC and highlight the complexity of the interaction between *C. albicans* and vaginal lactobacilli. Understanding these interactions could prove essential for the development of new strategies for treating VVC.

## 1. Introduction

Vulvovaginal candidiasis (VVC) is the second most common cause of lower reproductive tract infections in women of childbearing age. It is estimated that 75% of all women will suffer from VVC at least once in their lifetime [1], and nearly 10% of them are bound to experience recurrence. Recurrent VVC (RVVC), defined as four or more episodes of infection every year, markedly affects the quality of life, considering the severity of symptoms and the resulting psychological stress [2,3]. *C. albicans* is the etiological agent of almost all cases of VVC [1,4]. *C. albicans* is a commensal fungus of the vaginal mucosa; several host-related features and behavioral risk factors can affect the onset of VVC, including pregnancy, hyperglycemia, immunosuppression, antibiotic or glucocorticoid therapies, genetic predisposition, as well as vaginal microbiota composition. Oral contraceptive use, intrauterine devices, some hygiene habits, clothing, and sexual practices are all considered to be major behavioral risk factors associated with VVC [5].

*C. albicans* pathogenicity is supported by a plethora of virulence factors, including its ability to form biofilms, morphological transition between yeast and hyphal forms, expression of adhesins and invasins on its cell surface, secretion of enzymes such as secreted aspartyl proteases and phospholipases, and production of candidalysin, a cytolytic peptide toxin [6,7].

The ability of *C. albicans* to develop a complex biofilm on abiotic and biotic surfaces plays a key role in its pathogenicity [8]. Contrary to biofilms of other *Candida* spp., those formed by *C. albicans* display a more heterogeneous organization, formed by budding yeasts, a multilayer structure, and filamentous cells such as true hyphae and pseudohyphae, surrounded by extracellular matrix. The ability to form hyphae is critical for the development and maintenance of *C. albicans* biofilms. Indeed, hyphae are essential to supporting the structural integrity of biofilms and to provide a scaffold for the attachment of other yeast cells, pseudohyphae, as well as bacteria in polymicrobial biofilms [8,9,10].

Several studies have proposed that biofilm formation by *C. albicans* plays a crucial role in the development of vaginal candidiasis [11,12,13,14,15]. Wu et al. [16] supported the essential role of biofilm formation in the pathogenesis of vaginal candidiasis, demonstrating that histological damages of mucosal epithelial cells and local inflammation are associated with biofilm growth on the vaginal epithelium. In addition, it has been reported that *C. albicans* biofilm promotes the formation of persister cells, which are mainly responsible for the recalcitrance of vaginal candidiasis to antifungal drugs [14,17].

*Lactobacillus* spp. play a key role in maintaining vaginal health [18] and a decrease in their population, in particular of *L. crispatus*, is an important risk factor for the onset of vaginal infections [19,20]. However, recent studies have reported that not all *Lactobacillus* spp. are beneficial and protective in nature [21]. Tortelli et al. [22] showed that *L. iners* dominant communities were more permissive to vaginal colonization with potential pathogens such as *C. albicans.* Furthermore, Ceccarani et al. [20] demonstrated that VVC-positive women exhibited an increase in the relative abundance of *L. iners*, along with a decrease in that of *L. crispatus*.

*L. iners* is an unusual *Lactobacillus* species [23] with unique features. It shows a Gram-variable morphology, more complex nutritional requirements, produces few or no important protective factors, such as D-lactic acid and hydrogen peroxide; moreover, it is notable that it releases a cytolytic toxin [24]. *L. iners* dominance has been associated with adverse pregnancy outcomes [25,26] and infertility [27]. These characteristics have led to the hypothesis that *L. iners* can cause vaginal infections, including vaginal candidiasis [21,22,28]. Based on these assumptions, herein our main aim was to investigate the in vitro effect of *L. iners* cell-free supernatant (CFS) on the ability of *C. albicans* vaginal isolates to form biofilms. Further, we investigated the effects of *L. iners* CFS on biofilm cellular morphology, as well as on the expression of hyphae-associated genes.

## 2. Materials and Methods

### 2.1. Subjects

Fourteen nonpregnant, nondiabetic women aged between 18 and 48 years were enrolled at the Laboratory of Microbiology of the University Hospital “Santa Maria della Misericordia” (Perugia, Italy). Prior to enrollment, each of them completed a questionnaire indicating their health status and current symptoms of vaginal disease. All women signed an informed consent in accordance with the Declaration of Helsinki. This study was approved by the local ethical committee (Comitato Etico delle Aziende Sanitarie, Umbria, Italy) (CA 2020, 3802/19). All methods were performed in accordance with relevant guidelines and regulations. Each woman presented at least two of the following acute VVC signs and symptoms: vaginal discharge, itching, burning, and dyspareunia. Vaginal wet mount preparations were used to determine the number of polymorphonuclear cells per microscopic field, presence or absence of lactobacilli, and presence or absence of yeast and pseudohyphae/hyphae. The absence of other possible etiological agents responsible for vaginal infections was assessed by microbiological analysis.

### 2.2. Collection of C. albicans Isolates

All details pertaining to sample collection methods have been previously described [29,30]. Briefly, vaginal swabs from each patient were plated on CHROMagar Candida (VWR International P.B.I, Milan, Italy) and incubated at 37 °C for 48 h under aerobic conditions. Routine methods were used to identify yeast colonies, followed by confirmation with matrix-assisted laser/desorption ionization time-of-flight mass spectrometry (MALDI-TOF, bioMèrieux, Marcy-l’Étoile, France). All *C. albicans* strains were stored at −80 °C.

### 2.3. Microorganisms, Growth Media, and Growth Conditions

*C. albicans* ATCC 10231 and *L. iners* ATCC 55195 were purchased from the American Type Culture Collection (ATCC). *C. albicans* clinical isolates and reference strain were cultured on yeast extract peptone dextrose (YEPD) agar plates at 30 °C under aerobic conditions. *L. iners* ATCC 55195 was routinely grown in New York City (NYC) III broth at 37 °C for 24 h under anaerobic conditions. All microbial strains were freshly grown from frozen glycerol stocks (kept at −80 °C) before each experiment.

### 2.4. Growth Curve and L. iners CFS Preparation

An overnight NYC III broth culture of *L. iners* ATCC 55195 was 1:100 diluted in brain heart infusion (BHI) broth and incubated at 37 °C for 48 h under anaerobic conditions. At different timepoints (0, 2, 4, 6, 24, 28, 32, 48, and 52 h), 100 µL aliquots were withdrawn and diluted for CFU determination on NYC III agar plates, as previously described [31].

*L. iners* CFS was obtained at the end of exponential and decline growth phases. Briefly, after 24 h or 48 h of incubation, bacterial suspensions were centrifuged at 4000× *g* for 10 min, and the supernatant was aseptically decanted and sterilized using a syringe filter with a pore size of 0.45 µm. The pH of the supernatant was measured before storing it at −20 °C. Aliquots of *L. iners* CFS were immediately thawed before each experiment.

### 2.5. Biofilm Formation and Biomass Quantification

Biofilm formation was investigated as previously described by Gulati et al. [32]. Single colonies of *C. albicans* strains cultured on YEPD plates were inoculated into 5 mL of YEPD broth, followed by incubation at 30 °C for overnight, and cell densities were then assessed by measuring absorbance at OD_600_. The correlation between OD_600_ and CFU/mL was obtained by constructing a standard curve. Yeast cultures were then diluted with RPMI 1640 (with L-glutamine and 34.5 g/L MOPS, w/o sodium bicarbonate; pH 7) at 1 × 10^7^ cells/mL, and 200 µL was transferred to each well of a 96-well microtiter plate (Corning Inc., New York, NY, USA). The plates were then incubated at 37 °C for 1.5 h under aerobic conditions. Subsequently, the medium was discarded, and the wells were washed twice with 200 µL PBS to remove nonadherent cells. The culture medium was replaced with 200 µL of fresh medium, and the plates were incubated again for 24, 48, and 72 h under the same conditions.

Biofilm biomass was quantified using the crystal violet (CV) staining method, as previously described [31,32]. After incubation, the broth was discarded, and the wells were air dried for 45 min. After washing twice with 200 µL PBS, biofilms were stained with 110 µL of 0.4% CV for 45 min, and then washed four times with 200 µL distilled water. The CV bound to the biofilm was solubilized by adding 200 µL of 95% ethanol, followed by incubation for 45 min. Absorbance was measured at OD_590_ using a 96-well microplate reader (Tecan, Männedorf, Switzerland). Biofilm formation capability was determined as reported by Stepanovic et al. [30]. Clinical isolates were categorized into weak, moderate, or strong biofilm producers based on the calculated OD values for each strain and negative controls.

### 2.6. Biofilm Metabolic Activity Assessment

Biofilm metabolic activity was assessed by performing the 2,3-bis-(2-methoxy-4-nitro-5-sulfophenyl)-2H-tetrazolium-5-carboxanilide (XTT) reduction assay, as previously described [32]. Fresh XTT (Invitrogen, Waltham, MA, USA) was prepared in PBS at a final concentration of 0.5 mg/mL and stored at −80 °C. A phenazine methosulphate (PMS) solution was prepared at 0.32 mg/mL in sterile water. After biofilm incubation and cleaning, 200 µL of XTT–PMS solution (9:1 ratio of XTT and PMS) was added to each well, followed by incubation in the dark for 2 h at 37 °C under aerobic conditions. Absorbance was then measured at 492 nm using a 96-well microplate reader (Tecan, Männedorf, Switzerland).

### 2.7. Effect of L. iners CFS on C. albicans Biofilm Formation

The effect of *L. iners* CFS on *C. albicans* biofilm development was assessed according to the protocol described above. RPMI 1640 was mixed with *L. iners* CFS in a 1:1 ratio and added to *Candida* cultures both during the early adhesion and late maturation stages of biofilm development. In case of the control groups, biofilms were grown in RPMI 1640 mixed with fresh BHI broth. After 24 h of incubation, biofilm biomass and metabolic activity were determined, as previously described, and aliquots of CFS were used for pH determination.

### 2.8. Effect of L. iners CFS on C. albicans Biofilm Morphology

*C. albicans* clinical isolates and reference strain were incubated with *L. iners* CFS for 24 h, and after CV staining, they were microscopically analyzed with an inverted light microscope (Eurotek by Orma, Milan, Italy). Images were captured using a digital camera (200× and 400× magnifications).

### 2.9. Quantitative Analysis of Genes Associated with C. albicans Hyphal Formation

Total RNA was extracted from *C. albicans* ATCC 10231 and four clinical isolates, and the expression of hyphal-specific genes was determined. Briefly, total RNA was extracted from *C. albicans* biofilm cultures using TRIzol (Invitrogen, Waltham, MA, USA) and retrotranscribed into cDNA using the PrimeScript™ RT Reagent Kit with gDNA Eraser (Perfect Real Time) (Takara Bio Inc., Kusatsu, Japan). Quantitative real-time PCR (qRT-PCR) was performed using the QuantStudio 1 Real-Time PCR System (Applied Biosystems, Waltham, MA, USA). The analysis of *ACT1*, *ECE1* (extent of cell elongation 1), and *HWP1* (hyphal wall protein 1) expression levels was performed using SYBR Green Master Mix (Thermo Fisher Scientific, Waltham, MA, USA). Primer sequences were the same as those previously reported [29]. Real-time PCR was performed using the following cycling conditions: 3 min at 95 °C and 40 cycles of 10 s at 95 °C, 30 s of annealing at specific primer temperatures, and 45 s at 72 °C. Ct values of target genes were normalized on the *ACT1* housekeeping gene to obtain ΔCt, while ΔΔCt was obtained as the difference between the average ΔCT of the treated sample and the untreated sample. Relative changes in gene expression from qRT-PCR experiments were analyzed using the 2^−ΔΔCt^ method.

### 2.10. Statistical Analysis

All analyses were performed with Prism GraphPad 7. Values represent mean ± SEM of at least three independent experiments performed in triplicate. Data with normal distribution were analyzed using paired Student’s *t*-test or one-way analysis of variance and Dunnett’s multiple comparison test. For nonparametric variables, Wilcoxon matched-pairs signed-rank test or Kruskal–Wallis test and Dunn’s multiple comparison test were performed. *p* < 0.05 indicated statistical significance.

## 3. Results

### 3.1. Growth Curve of L. iners

**Figure 1:**
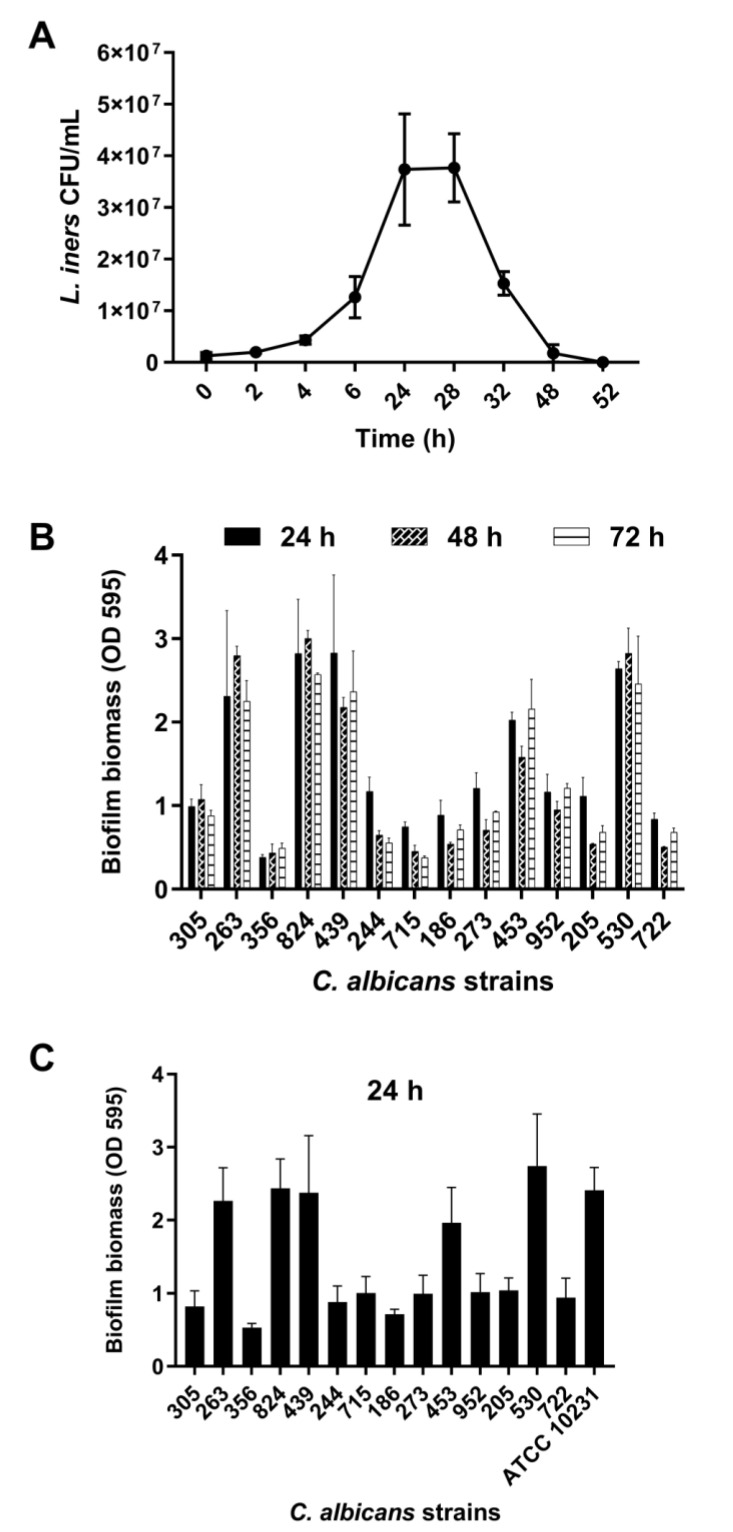
(**A**) Growth curve of *Lactobacillus iners*. *L. iners* ATCC 55195 was incubated for 52 h in brain heart infusion (BHI) broth, and bacterial growth was determined at selected time points. Values represent mean ± SEM of colony-forming units (CFU)/mL from three independent experiments performed in duplicate. (**B**) Biofilm formation by *Candida albicans* clinical isolates in vitro. The isolates were grown for 24, 48, and 72 h. Biofilm biomass was evaluated using the crystal violet staining method, and values represent mean ± SEM of absorbance at OD_595_. Data are from at least three independent experiments with *n* = 6. (**C**) Biofilm formation by *C. albicans* clinical isolates in vitro in RPMI 1640 + BHI broth. Biofilms were grown for 24 h using 1:1 RPMI 1640 and BHI broth. Values represent mean ± SEM of absorbance at OD_595_ from at least three independent experiments with *n* = 6.

*L. iners* is an unusual member of the human vaginal microbiota and does not show growth on de Man–Rogosa–Sharpe medium, which is normally used to isolate vaginal lactobacilli. To obtain CFS, *L. iners* was grown in BHI broth. As evident from Figure 1A, *L. iners* showed the ability to adapt and grow in BHI media after approximately 3 h of incubation.

The growth curve exhibits the exponential growth phase from 6 h to 24 h of incubation, followed by the stationary growth phase, lasting for 4 h, and ultimately the decline growth phase, beginning 28 h after incubation. CFS was recovered at the end of both the exponential (24 h CFS) and decline (48 h CFS) phases of growth. The average pH value was 6.0 (5.5–6.4) for both 24 and 48 h CFS.

### 3.2. Biofilm Formation by Vaginal Isolates of C. albicans

Fourteen clinical isolates of *C. albicans* obtained from women with VVC were analyzed for their ability to form biofilms under our experimental conditions. These isolates (1 × 10^7^ CFU/mL) were incubated in flat-bottomed 96-well microtiter plates in RPMI 1640 at 37 °C for 24, 48, and 72 h, followed by biofilm biomass quantification. We found that all strains formed a mature biofilm within 24 h of incubation, with a clear heterogeneity between them (Figure 1B). Based on biofilm biomass quantification, the isolates were categorized into strong, moderate, or weak biofilm producers [33]. Eight isolates (305, 244, 715, 186, 273, 952, 205, 722) were moderate biofilm producers (0.60 < OD ≤ 1.21), five isolates (263, 824, 439, 453, 530) were strong biofilm producers (OD > 1.21), and one isolate (356) was a weak biofilm producer (OD ≤ 0.60). Biofilm formation was also determined in 50% RPMI 1640–50% BHI media after 24 h of incubation at 37 °C (Figure 1C). We observed that the presence of BHI did not influence the ability of *C. albicans* to form biofilms. Furthermore, in these experimental conditions, *C. albicans* ATCC 10231 showed the ability to produce a strong biofilm.

### 3.3. Effects of L. iners CFS on C. albicans Biofilm Formation

**Figure 2:**
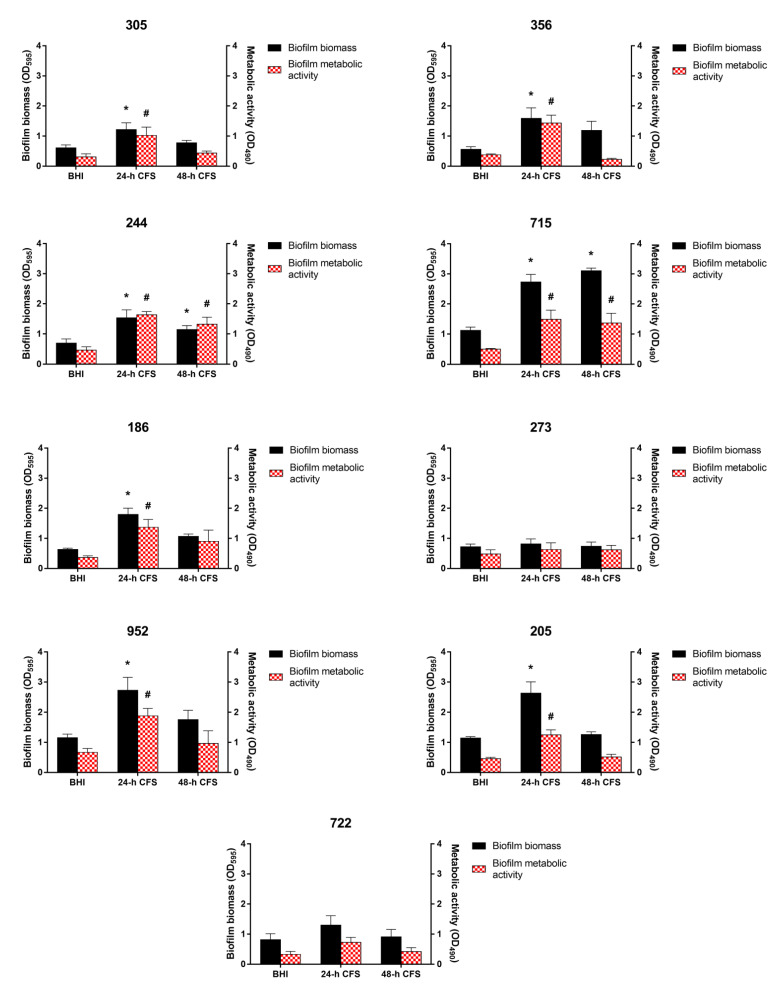
Effect of *Lactobacillus iners* cell-free supernatant (CFS) on in vitro biofilm formation by *Candida albicans* clinical isolates characterized as moderate (305, 244, 715, 186, 273, 952, 205, 722) and weak (356) biofilm producers. Biofilms of *C. albicans* clinical isolates were grown for 24 h using RPMI 1640 + BHI broth or 24/48 h *L. iners* CFS in a 1:1 ratio. Biofilm biomass was evaluated using the crystal violet staining method (OD_595_, black bars, left y-axis), and metabolic activity was determined using the XTT reduction assay (OD_490_, red bars, right y-axis). Values represent mean ± SEM of at least three independent experiments performed in triplicate. Statistically significant differences were tested using one-way analysis of variance or Kruskal–Wallis test. * *p* < 0.05 biomass of biofilm grown with 24/48 h *L. iners* CFS vs. control biofilm biomass (BHI). ^#^ *p* < 0.05 metabolic activity of biofilm grown with 24/48 h *L. iners* CFS vs. control biofilm metabolic activity (BHI).

To evaluate the effects of *L. iners* CFS on *C. albicans* biofilm formation, 100 µL of 24 or 48 h CFS in BHI was mixed with 100 µL of each *C. albicans* isolate and *C. albicans* ATCC 10231 reference strain (1 × 10^7^ CFU/mL) in RPMI 1640, followed by incubation for 24 h, as described earlier. Biofilm quantification was performed by biofilm biomass and metabolic activity determination. As is evident from Figure 2, seven of the nine moderate or weak biofilm producers displayed a significant increase in biofilm biomass and metabolic activity in the presence of *L. iners* 24 h CFS, whereas only two isolates (244 and 715) showed an increase in these parameters in the presence of *L. iners* 48 h CFS. 

**Figure 3:**
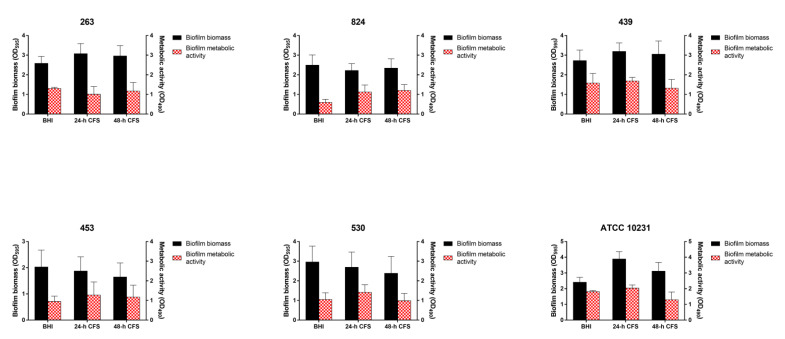
Effect of *Lactobacillus iners* cell-free supernatant (CFS) on in vitro biofilm formation by *Candida albicans* clinical isolates characterized as strong biofilm producers and *C. albicans* ATCC 10231 reference strain. Biofilms of *C. albicans* clinical isolates and reference strain were grown for 24 h using RPMI 1640 + BHI broth or 24/48 h *L. iners* CFS in a 1:1 ratio. Biofilm biomass was evaluated using the crystal violet staining method (OD_595_, black bars, left y-axis), and metabolic activity was determined using the XTT reduction assay (OD_490_, red bars, right y-axis). Values represent mean ± SEM of at least three independent experiments performed in triplicate. Statistically significant differences were tested using one-way analysis of variance or Kruskal–Wallis test.

In contrast, *L. iners* 24 or 48 h CFS had no effects on the ability of strong biofilm producers to form biofilms (Figure 3).

We also determined the pH of *C. albicans* biofilms developed in the absence or presence of *L. iners* 24 h CFS. *C. albicans* biofilm cultures showed a pH value of 6.4, indicating that pH was not significantly modulated and maintained near neutral.

### 3.4. Effects of L. iners CFS on C. albicans Biofilm Morphology

**Figure 4:**
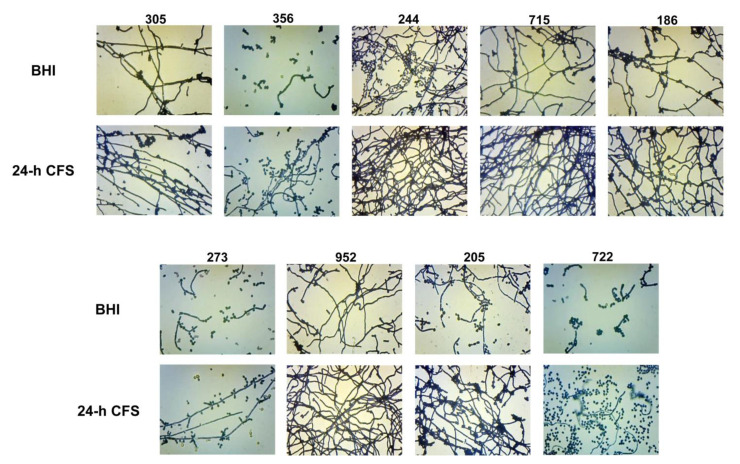
Effect of *Lactobacillus iners* cell-free supernatant (CFS) on biofilm morphology of *Candida albicans* clinical isolates characterized as moderate (305, 244, 715, 186, 273, 952, 205, 722) and weak (356) biofilm producers. Biofilms of *C. albicans* clinical isolates were grown for 24 h using RPMI 1640 + BHI broth or 24 h *L. iners* CFS in a 1:1 ratio and stained with crystal violet. Images of biofilms were acquired with an inverted light microscope at 200×. Representative microscopic images from three different experiments are shown.

The yeast-to-hyphal transition is a key feature of biofilm formation by *C. albicans.* Herein we investigated the effects of *L. iners* 24 h CFS on *C. albicans* biofilm morphology. As shown in Figure 4, in comparison to respective controls, *L. iners* culture supernatants induced an increase in the growth of *C. albicans* filamentous forms by all moderate (305, 244, 715, 186, 273, 952, 205, 722) and weak (356) biofilm producers. In contrast, *L. iners* CFS did not affect hyphal growth in strong biofilm producers (clinical isolates or reference strain) (Supplementary Figure S1). 

**Figure 5:**
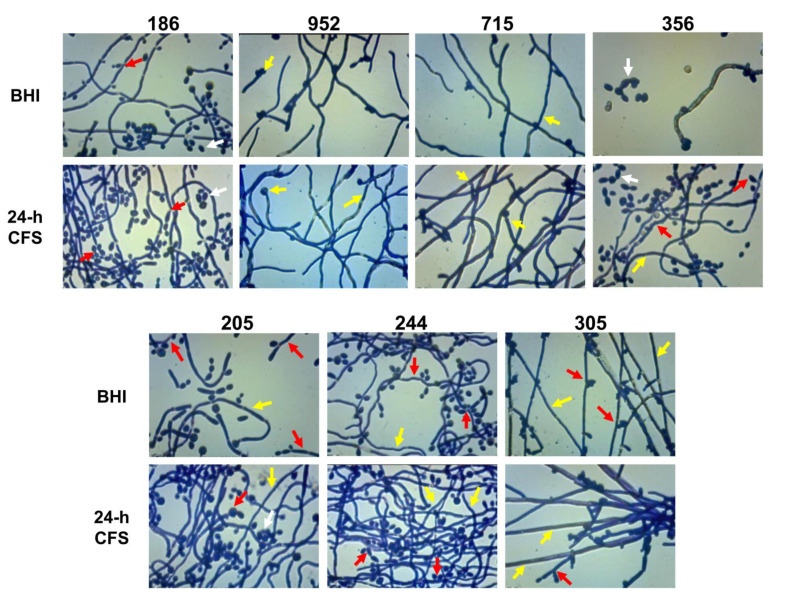
*Lactobacillus iners* cell-free supernatant (CFS) promotes filamentous growth by *Candida albicans*. The effect of *L. iners* CFS on hyphal/pseudohyphal growth of *C. albicans* characterized as moderate/weak biofilm producers was microscopically determined after crystal violet staining. Optical microscopy images (magnification 400×) of *C. albicans* isolates showed that the hyphae/pseudohyphae mass was significantly higher in the presence of *L. iners* CFS than in controls cultured in BHI. Red arrows show branched *C. albicans* pseudohyphae, which appear ellipsoidal in shape, pointing to constrictions at septal sites, bearing single or multiple lateral blastoconidia. Yellow arrows show well-structured biofilms mainly composed of hyphae characterized as unconstricted filaments with parallel-sided walls and true septa. Clusters of unicellular oval or spherical yeasts (white arrows), some of them replicating as budding daughter cells, are also shown.

The biofilm morphology of all the moderate/weak isolates (*n* = 7) which showed an increase in both biofilm formation as well as filamentous growth was further microscopically analyzed at magnification 400×. Relative to respective controls (BHI), in the presence of *L. iners* 24 h CFS, we observed that apart from the clinical isolate 186 that formed a biofilm predominantly composed of clusters of yeast cells and pseudohyphae, and the strains 952 and 715, which formed true hyphae, all the remaining strains tested produced biofilms that were composed of both the filamentous forms (Figure 5). In addition, for all moderate/weak biofilm producers, we investigated the effects of pH on pseudohyphal/hyphal growth induced by *L. iners* 24 h CFS. We observed *C. albicans* biofilm formation at pH 4.9. All tested strains failed to produce hyphae, and thus, resultant biofilms were composed of only clusters of yeast cells (data not shown).

### 3.5. L. iners Culture Supernatants Upregulated the Expression of C. albicans Hyphal-Specific Genes

Our previous studies have shown that two key hyphae-associated genes, *ECE1* and *HWP1* [34,35,36], were overexpressed during human vaginal candidiasis, and that their upregulation was associated with NLRP3 inflammasome activation, a crucial player in the immunopathogenesis of VVC [29,37]. Based on these results, here we analyzed their expression level to gain insights into the mechanism by which *L. iners* 24 h CFS can modulate the pseudohyphal/hyphal growth in *C. albicans*.

**Figure 6:**
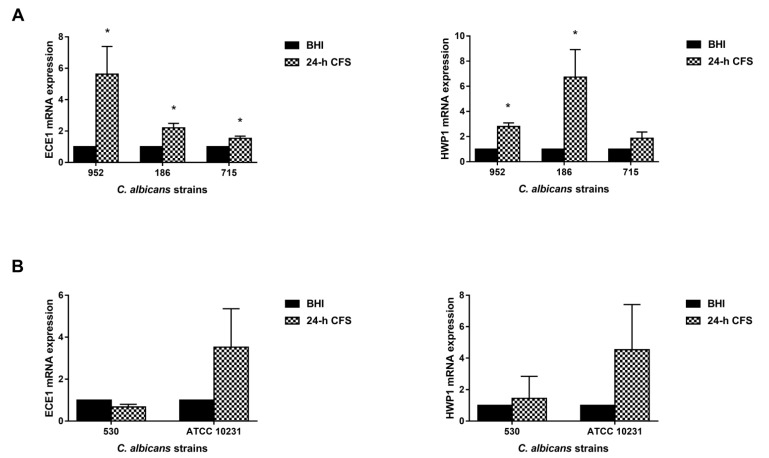
Effect of *Lactobacillus iners* 24 h cell-free supernatant (CFS) on the expression of hyphal-specific genes of *Candida albicans* clinical isolates and *C. albicans* ATCC 10231 reference strain. Total RNA was extracted from biofilms grown with or without 24 h *L. iners* CFS, retrotranscribed into cDNA, and real-time PCR was performed to assess expression of *ACT1*, *ECE1*, and *HWP1*. (**A**) Relative gene expression levels of *ECE1* and *HWP1* of three *C. albicans* clinical isolates characterized as moderate biofilm producers and (**B**) one characterized as strong biofilm producer with the ATCC 10231 reference strain. Data were analyzed using the 2^−ΔΔCt^ method, and values represent mean ± SEM of three (panel A) and two (panel B) independent experiments performed in triplicate. Statistically significant differences were tested with paired Student’s *t*-test or Wilcoxon matched-pairs signed-rank test. * p < 0.05 relative gene expression of 24 h *L. iners* CFS-treated biofilms vs. control biofilms.

Three moderate biofilm producers were chosen: the isolate 186 forming only pseudohyphae and the 952 and 715 forming only true hyphae. Transcriptional levels were quantified by qRT-PCR. The expression level of each gene was normalized with that of a housekeeping gene (*ACT1*) for 24 h CFS-treated as well as -untreated *C. albicans* biofilms; data are presented as relative expression fold change. In comparison with the control, *L. iners* 24 h CFS significantly upregulated the expression levels of *HWP1* and *ECE1* in all three and two of the three moderate biofilm producers, respectively (Figure 6).

Transcriptional levels were also analyzed in two strong biofilm producers (one clinical isolate, 530, and the reference strain ATCC 10231), but *L. iners* CFS did not significantly modulate the expression levels of *HWP1* or *ECE1* in them.

## 4. Discussion

*C. albicans* is a common commensal fungus of the vaginal mucosa and also a pathogen that is responsible for almost 90% of all VVC cases [38]. It was recently reported that VVC-positive women present a depletion in the populations of health-associated *Lactobacillus* spp., such as *L. crispatus*. A decrease in the population of *L. crispatus* is associated with an increase in that of *L. iners* [13,20], an unusual *Lactobacillus* species that does not seem to have a protective effect against VVC [23,28]. Deciphering the interaction between *C. albicans* and *L. iners* is crucial for increasing our knowledge of *C. albicans* pathogenicity and for developing new diagnostic tools and effective therapeutic approaches.

The ability of *C. albicans* to produce biofilms is considered to be one of the most important determinants of this common gynecological disease [38] and a critical factor responsible for conferring resistance to antimycotic drugs, such as fluconazole and amphotericin B [11,39,40,41], and consequently for RVVC development. To the best of our knowledge, we investigated, for the first time, the in vitro effects of *L. iners* CFS on the biofilm formation ability of *C. albicans* vaginal isolates. We found that *L. iners* CFS enhanced the biofilm-formation ability of several *C. albicans* isolates. According to previous studies [13,14,41], vaginal isolates display an ability to form mature, heterogeneous biofilms. We observed that *L. iners* CFS induced a significant increase in both biofilm biomass and metabolic activity of 77.7% of *C. albicans* clinical isolates that were moderate or weak biofilm producers, transforming them from moderate or weak biofilm producers to strong biofilm producers and consequently increasing their virulence. Indeed, clinical isolates capable of forming robust, well-structured biofilms are more pathogenic than weak biofilm producers [41,42]. These data suggest that *L. iners*-dominated vaginal microbiome can not only contribute to the onset of VVC, but also affect the management of such infections, increasing the probability of recurrence. McKloud et al. [13] suggested that women experiencing RVVC for >6 months show an imbalance of the vaginal microbiota, characterized by an abundance of *L. iners* and a reduction in health-associated *Lactobacillus* spp. Besides, McKloud et al. [13] demonstrated that *L. iners* was unable to modulate biofilm formation by *C. albicans*. One reason for this apparent discrepancy with our data could be differences in experimental conditions. We studied the effects of *L. iners* CFS on the biofilm formation ability of *C. albicans* clinical isolates, whereas McKloud et al. used reference strains of *L. iners* and *C. albicans* (DSMZ 13,335 and 5314, respectively). Furthermore, McKloud et al. used *C. albicans* and *L. iners* cocultures, instead of *L. iners* CFS.

The ability to produce filamentous forms is critical for the development and maintenance of *C. albicans* biofilms [8,10,43,44]. We found that *L. iners* CFS enhanced the growth of the filamentous forms only in those strains that became strong biofilm producers from moderate or weak biofilm producers. Our findings are consistent with those of previous studies, indicating a direct relationship between the ability of *C. albicans* to form biofilms and pseudohyphal/hyphal growth [45,46,47]. Therefore, it is possible that *L. iners-*dominated vaginal microbiome favors the formation of robust, stable *C. albicans* biofilms on the vaginal mucosa as well as on implanted devices, such as intrauterine devices [48], which is one of the most used methods to prevent fertilization [49]. It is well-established that pH plays a key role in the yeast-to-hyphal morphogenetic transition [35,50]; neutral pH provides a permissive environment for hyphal formation [51], while low pH prevents hyphal formation, thus promoting the growth of fungus as yeast cells [52]. Our data showed that the pH of *C. albicans* biofilms was near neutral, and that the presence of *L. iners* CFS, differently to other lactobacilli [53,54,55], did not reduce the pH value. This could be because *L. iners* produces less lactic acid than *L. crispatus*, and lactic acid is the main acidifier in the vaginal environment [56,57]. Considering that *C. albicans* is capable of alkalinizing the external environment [51], it is possible that in vaginal econiches where *C. albicans* coexists with *L. iners*, the pH is less acidic or even near neutral.

To elucidate the potential mechanism underlying the ability of *L. iners* to induce the growth of filamentous forms of *C. albicans*, we also assessed the expression levels of *HWP1* and *ECE1*, which encode proteins essential for hyphal formation and play a key role in, at least, two phases of VVC pathogenesis: adhesion and tissue damage. In particular, *HWP1* is crucial for *C. albicans* adhesion; it promotes its binding to epithelial cells, enabling colonization [58,59] and biofilm formation [12,60,61,62]. *ECE1* is highly expressed by hyphae during the invasion of epithelial cells. It encodes candidalysin, a toxin recently identified by Moyes et al. [7], which directly damages host epithelial membranes, triggering a danger-response signaling pathway with consequent activation of the epithelial immune response [63]. Our results showed that *L. iners* CFS upregulated the expression of both *HWP1* and *ECE1*. These results are in line with those of our previous studies, which reported an overexpression of these genes in vaginal swabs of symptomatic women with VVC [29,37]. The limitations of this study are the small number of *C. albicans* clinical isolates tested and the use of *L. iners* CFS instead of live bacteria, which could be more representative of all the interactions occurring in vivo. A further limitation is that of not having tested supernatants from clinical isolates of *L. iners*. Their use would be helpful in confirming our data.

Overall, our findings demonstrate that *L. iners* promotes *C. albicans* virulence by enhancing pseudohyphal/hyphal growth and biofilm formation by moderate and weak biofilm producers, implying that the presence of *L. iners* in the vaginal environment, unlike other *Lactobacillus* spp., might not be a good indicator of vaginal health. Further in vitro and in vivo studies are warranted on this intriguing topic.

## Data Availability

The datasets used and/or analyzed during the current study are available from the corresponding author on reasonable request.

## Supplementary Materials

The following are available online at https://www.mdpi.com/article/10.3390/microorganisms9122577/s1. Figure S1. Effect of *Lactobacillus iners* cell-free supernatant (CFS) on biofilm morphology of *Candida albicans* clinical isolates characterized as strong biofilm producers.
