# Supplementary material for: Lactobacillus iners Cell-Free Supernatant Enhances Biofilm Formation and Hyphal/Pseudohyphal Growth by Candida albicans Vaginal Isolates
Source: Microorganisms. 2021 Dec 13;9(12):2577. doi: 10.3390/microorganisms9122577 (PMC8706969; doi:10.3390/microorganisms9122577)
Supplement: Supplementary file 1 [file microorganisms-09-02577-s001.zip › microorganisms-1487455-supplementary.pdf]

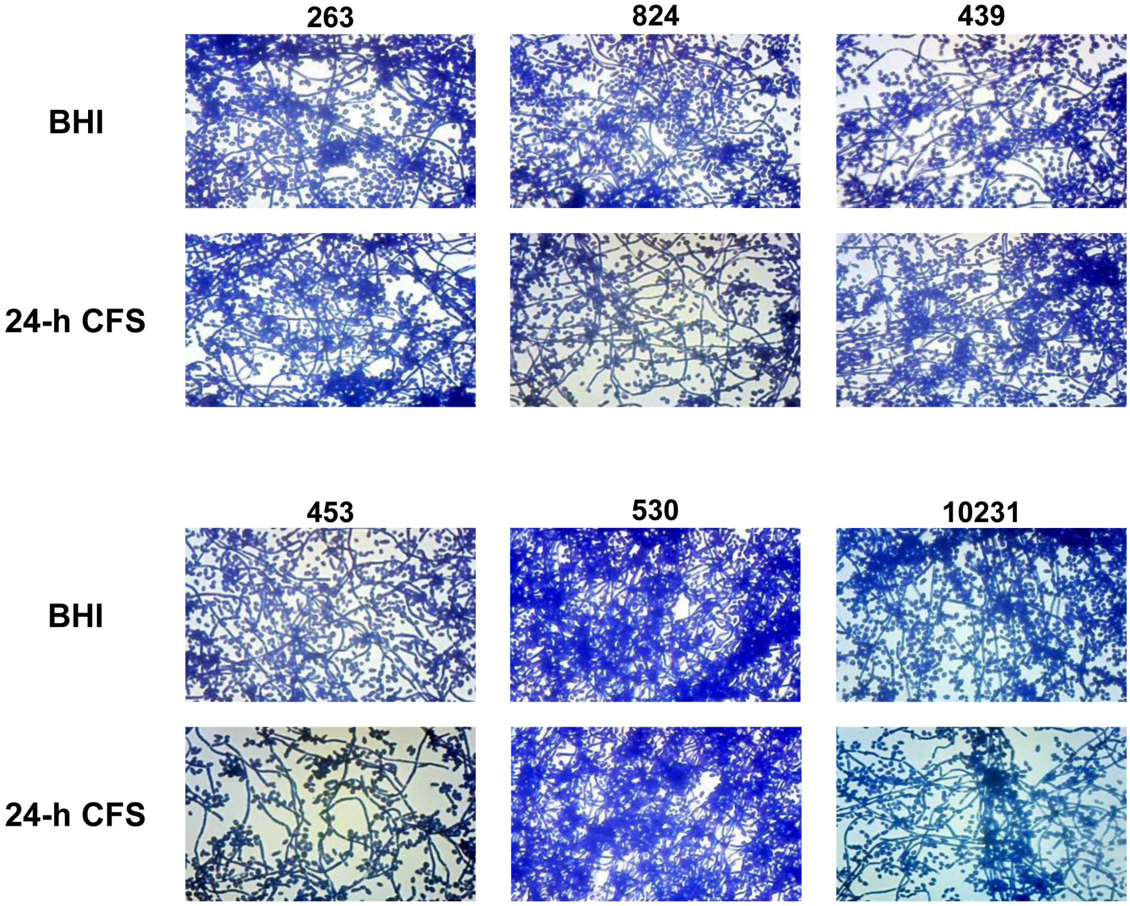

Figure S1. Effect of *Lactobacillus iners* cell-free supernatant (CFS) on biofilm morphology of *Candida albicans* clinical isolates characterized as strong biofilm producers.
